# Development of alternative splicing signature in lung squamous cell carcinoma

**DOI:** 10.1007/s12032-021-01490-1

**Published:** 2021-03-27

**Authors:** Jia-qing Yan, Min Liu, Ying-lin Ma, Kai-di Le, Bin Dong, Guo-hui Li

**Affiliations:** Department of Pharmacy, National Cancer Center/National Clinical Research Center for Cancer/Cancer Hospital, Chinese Academy of Medical Sciences and Peking Union Medical College, Beijing, 100021 China

**Keywords:** Lung squamous cell carcinoma, Alternative splicing, Splicing factor, LASSO, Signature

## Abstract

Increasing evidence demonstrated that alternative splicing (AS) plays a vital role in tumorigenesis and clinical outcome of patient. However, systematical analysis of AS in lung squamous cell carcinoma (LUSC) is lacking and greatly necessary. Thus, this study was to systematically estimate the function of AS events served as prognostic indicators in LUSC. Among 31,345 mRNA AS events in 9633 genes, we detected 1996 AS in 1409 genes which have significant connection with overall survival (OS) of LUSC patients. Then, prognostic model based on seven types of AS events was established and we further constructed a combined prognostic model. The Kaplan–Meier curve results suggested that seven types of AS signatures and the combined prognostic model could exhibit robust performance in predicting prognosis. Patients in the high-risk group had significantly shorter OS than those in the low-risk group. The ROC showed all prognostic models had high accuracy and powerful predictive performance with different AUC ranging from 0.837 to 0.978. Moreover, the combined prognostic model had highest performance in risk stratification and predictive accuracy than single prognostic models and had higher accuracy than other mRNA model. Finally, a significant correlation network between survival-related AS genes and prognostic splicing factors (SFs) was established. In conclusion, our study provided several potential prognostic AS models and constructed splicing network between AS and SFs in LUSC, which could be used as potential indicators and treatment targets for LUSC patients.

**Supplementary Information:**

The online version contains supplementary material available at 10.1007/s12032-021-01490-1.

## Introduction

Lung cancer is one of the common human malignancies and is the leading cause of cancer-associated deaths worldwide, resulting in approximately 1.7 million deaths (18.4%) worldwide in 2018 [1] and about 140,000 deaths per year in the USA in 2019 and 2020 [2, 3]. The non-small cell lung cancer (NSCLC) mainly included lung adenocarcinoma (LUAD), lung squamous cell carcinoma (LUSC), and large cell lung carcinoma (LCLC) [4]. LUSC accounts for approximately 30% of all lung cancer cases [4]. Unfortunately, Patients tend to be older, typically diagnosed at advanced stage, and lack of effective molecular-targeted drugs [5]. Thus, they have a poor prognosis with five-year survival rate of < 15% [6]. Currently, the pathogenesis and progressive mechanisms of LUSC remain unclear. Though great progress has been made in diagnosis and targeted treatment of lung cancer, its clinical outcome is still unsatisfactory [7–9]. Therefore, it is necessary to further systematically understand the biological mechanisms in LUSC, which could facilitate the development of target therapy and prognostic biomarkers of LUSC patients.

Alternative splicing (AS) is the process of removing introns from most human multi-exon genes and alternatively including or excluding specific exons. AS of pre-mRNA is one of the most extensive and sophisticated mechanisms to interpret proteome diversity and produce mature mRNAs and protein variants structurally and functionally [10]. In addition to protein diversity, the translation of mRNA isomer was also downregulated by AS events through resulting in degradation of premature stop codon [11]. Hence, AS was an indispensable procedure and alterations in splicing patterns had a close relationship with protein functions. In the past few years, extensive genomic and functional investigations had found that initiation of specific isoforms and splicing defects were driving factors for cancer [12, 13]. For the past few years, accumulating evidence has illustrated that unusual AS events could exert a straight role in the process of biogenesis and deterioration of cancers by involving in cell proliferation, migration, immune escape and other process [14].

Recently, many studies have described the perturbation of AS events in various cancer, including lung cancer. For example, *Kong-Beltran *et al. observed met exon 14 in lung cancer which could contribute to protein regions' deletion that restricts its kinase catalytic activity [15]. More recently, analyses of AS have also shown prognostic value for a variety of cancer types, including non-small cell lung cancer [7], ovarian cancer [16], breast cancer [17] and so on. Currently, several studies have mainly concentrated on identifying different AS events between cancer tissues and normal controls or prognostic AS events [18, 19]. Nevertheless, few articles systematically reported the LUSC-specific AS events associated with survival of patients.

Moreover, several studies have shown that alteration in splicing factors might facilitate to activate oncogenes and tumor pathways or alternatively destruct the effect of tumor suppressors [20, 21]. Hence, it is imperative to draw a comprehensive regulatory network of SFs [22, 23]. Considering the close connection between AS and SFs and the fact that they are only superficially understood, it is imperative to investigate their prognostic property, as well as the regulatory mechanism in LUSC.

The Cancer Genome Atlas (TCGA) project provides abundant resources, such as Exon, splice, and transcript isoform expression levels, to investigate AS patterns of cancers [24]. We systematically profiled the genome-wide LUSC-specific alternative splicing events from TCGA. The purpose of this study is to explore the roles of alternative splicing events that could be considered as prognostic biomarkers in LUSC. Findings in this study would contribute to exploit novel and appropriate therapeutic treatments for LUSC.

## Material and methods

### Data curation process

SpliceSeq data of TCGA-LUSC and corresponding clinical information were downloaded from the TCGA data portal (https://portal.gdc.cancer.gov/) [24]. SpliceSeq tool, a java-based application, was usually used to unambiguously quantify the mRNA splicing levels of samples in TCGA. A novel value could be calculated by SpliceSeq based on seven types of AS events about each protein-coding gene provided from Ensemble gene database [16, 25]. For the following seven kinds of AS events, the Percent Spliced In (PSI) value was calculated, quantifying splicing event levels are ranging from 0 to 1.

### Establishment of prognostic model

A total of 487 LUSC patients were included in this study (Table S1). The PSI value of AS events in samples were collected and subjected to univariate Cox analysis. All the AS events were screened out *having P* value < 0.05, and these events was considered as a candidate prognosis-related events. We performed the least absolute shrinkage and selection operator (LASSO) Cox regression model using “glmnet R” package to select the most valuable and concise AS events in all AS events filtered in univariate Cox analysis (*P* < 0.05) and then constructed predictive models based on survival-related AS events by multivariate Cox analysis. Then, Based on the coefficient of each above AS event, each patients' risk score could be calculated by the signature, respectively. Meanwhile, all patients were divided into distinct subgroups based on the median value of risk scores.

### Survival analysis

The Kaplan–Meier curve was performed to analyze differential survival. The receiver operating characteristic curves (ROC) were conducted to explore sensitivity and specificity of prognostic signatures using “survivalROC” R package (https://www.r-project.org/, v.3.5.3). Univariate and multivariate Cox regression analysis were performed to investigate the prognostic independence of AS signature and clinical characteristics using forest plot R package.

### Upset plot and splicing factor-regulated network establishment

The Upset intersective plot, a more scalable visualizing diagrams to Venn, was used to explore the interactive sets of those AS events and “UpSet” R package was used to visualize their potential interrelationship. Expression data of the Splicing factors (SFs) were extracted from TCGA-LUSC mRNA-seq data. All SF genes were subjected to univariate Cox analysis and when their *P* < 0.05, these SFs were considered as the survival-associated splicing factors. The relationship closeness between SFs expression value and AS’s PSI value was calculated by spearman test. Meanwhile, a network of the interaction between these SFs and prognostic AS events was illustrated by Cytoscape 3.7.0 (https://cytoscape.org/).

## Results

### Overview of AS events in TCGA-LUSC

**Fig. 1:**
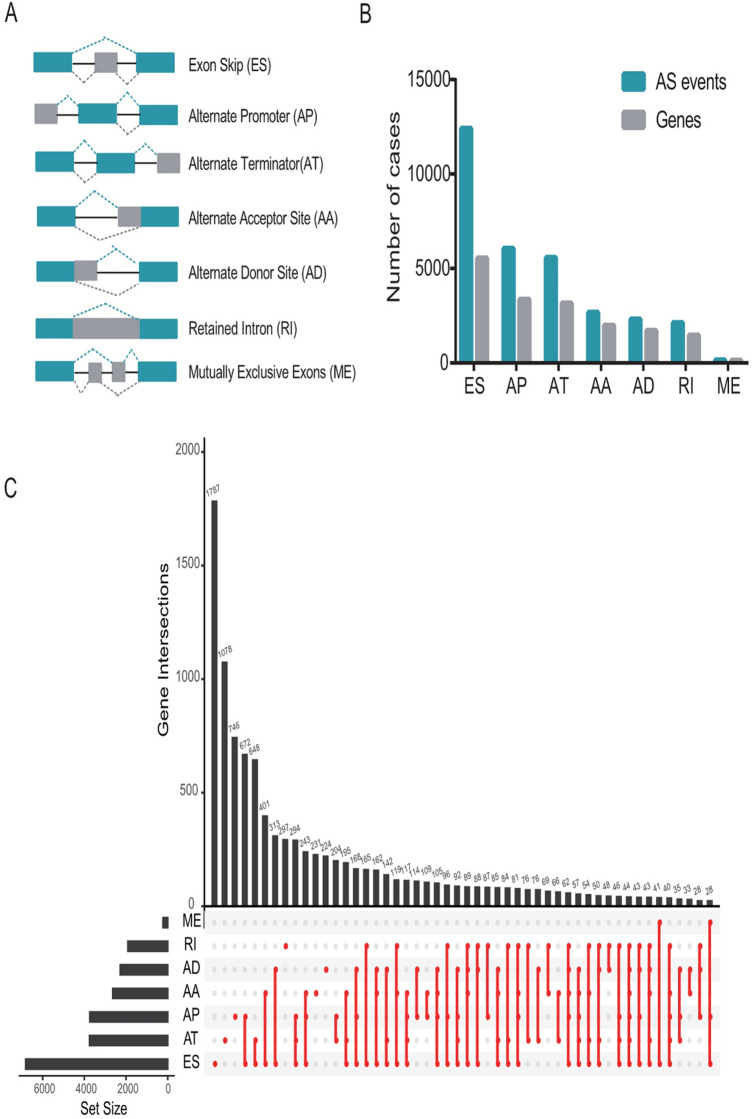
Overview of AS events in TCGA-LUSC cohort. **a** Seven types of AS events were illustrated including exon skip (ES), retained intron (RI), alternate promoter (AP), alternate terminator (AT), alternate donor site (AD), alternate acceptor site (AA), and mutually exclusive exons (ME). **b** Numbers of AS events and AS-associated genes in 487 LUSC patients. **c** UpSet plot of overlapping genes among the seven patterns of AS events

Integrated AS events were analyzed in 487 TCGA-LUSC data set (Fig. 1a). A total of 31,345 AS events from 9633 genes were detected including 12,416 ES in 5554 genes, 6073 AP in 3369 genes, 5579 AT in 3167 genes, 2675 AA in 1996 genes, 2323 AD in 1722 genes, 2129 RI in 1474 genes, and 150 ME in 144 genes (Fig. 1b). Among them, ES events were the most common type, accounting for more than one-third of all events, followed by AP and AT events, while ME was the least. Notably, the amount of AS events far exceeded the number of genes. Furthermore, a subset of overlapping AS events among the seven types of AS in LUSC was illustrated by UpSet plot diagram (Fig. 1c).

### Identification of prognosis-related AS events in LUSC

**Fig. 2:**
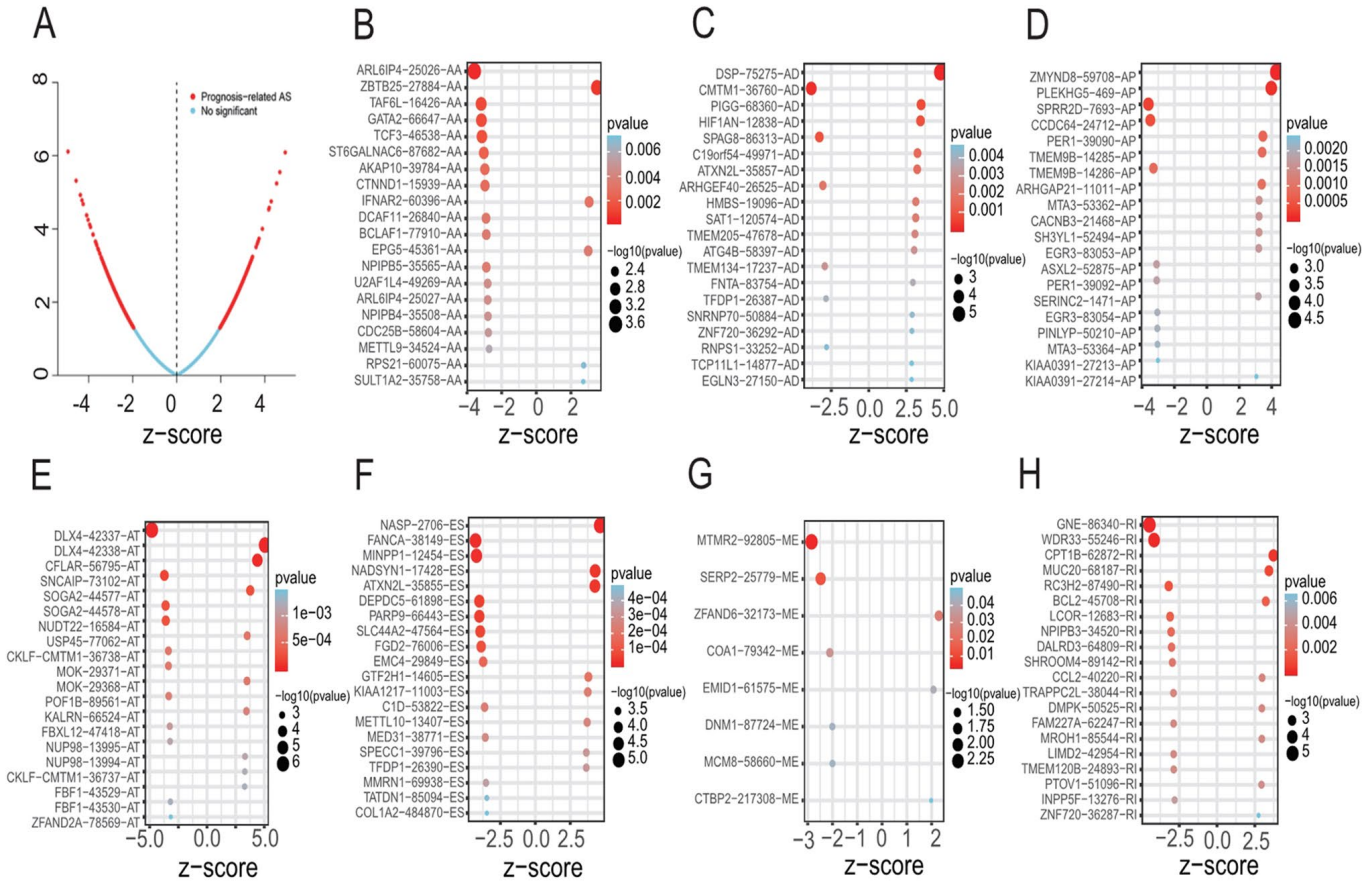
Forest plots analyses of survival-associated AS events. **a** Volcano plot depicting the *P* values from univariate Cox analysis of the 31,345 AS events. **b–h** Forest plots of z-score of the top 20 significantly survival-related AS events for seven splicing types (ME only eight events)

First of all, we conducted a univariate Cox analysis of the 31,345 AS events in 487 LUSC patients to evaluate the relationship between AS events and overall survival (OS) status in LUSC. Consequently, 1996 AS events within 1409 genes were obviously related to overall survival of LUSC patients (Fig. 2a). As shown in Fig. 2b–h, the top 20 AS events were significantly related to OS among seven types of AS events. Interestingly, some of survival-associated AS genes underwent multiple types of AS events. For example, AA, AD, RI, and ES of ATXN2L and AA, AP, and RI of NPIPB4 were conspicuously related to OS of LUSC patients.

### Establishment of prognostic AS signatures for LUSC patients

**Fig. 3:**
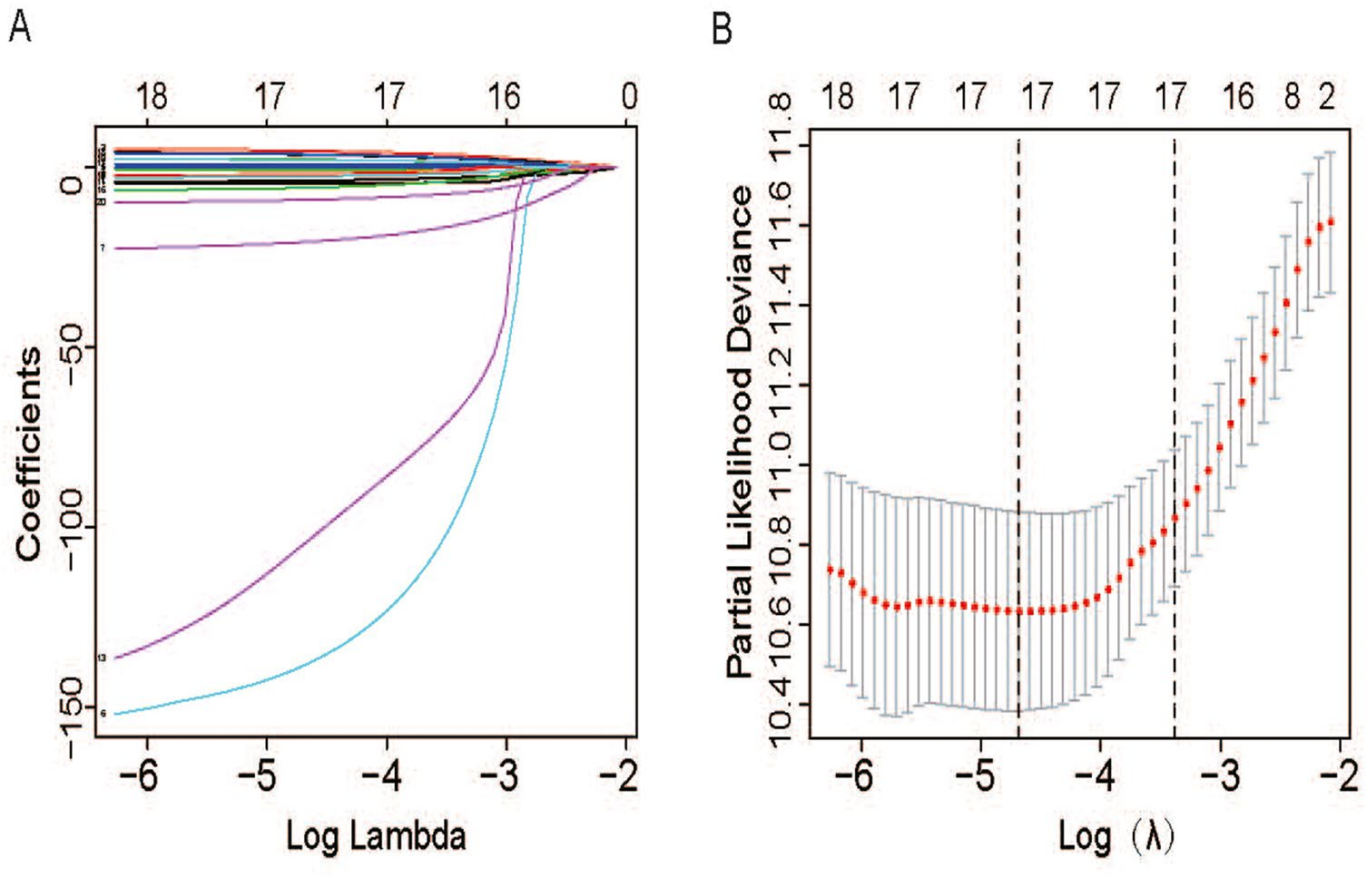
Survival-related AS events were selected using the LASSO Cox analysis. **a** LASSO coefficient profiles of the candidate survival-related AS events. **b** Dotted vertical lines were drawn at the optimal values by using the minimum criteria

**Fig. 4:**
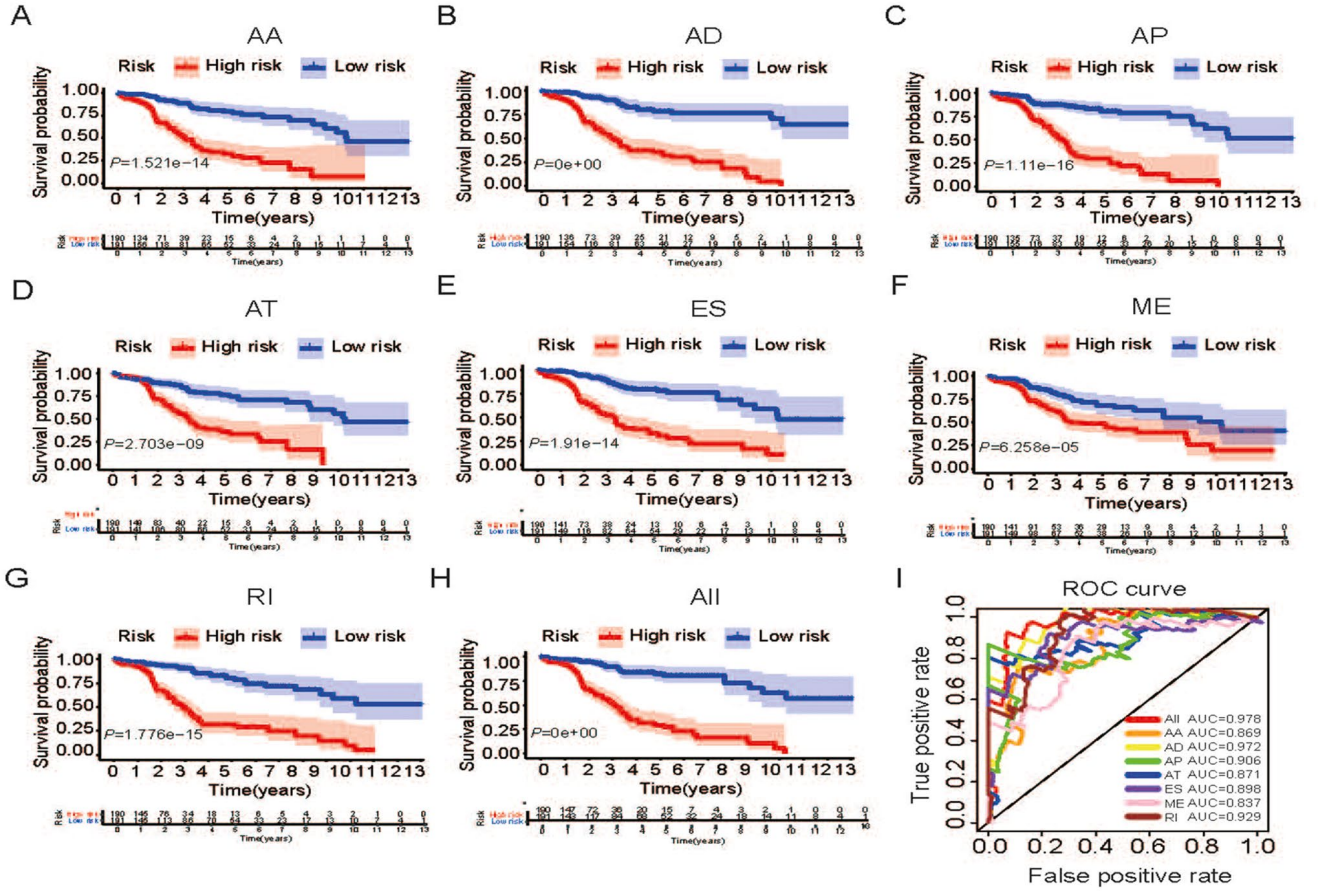
The Kaplan–Meier curves and ROC curves of prognostic AS models. **a–g** The Kaplan–Meier plots of seven types of AS events, respectively. **h** The Kaplan–Meier plots of combined prognostic model. **i** The ROC curves for overall survival of seven types of AS events and combined prognostic model

**Fig. 5:**
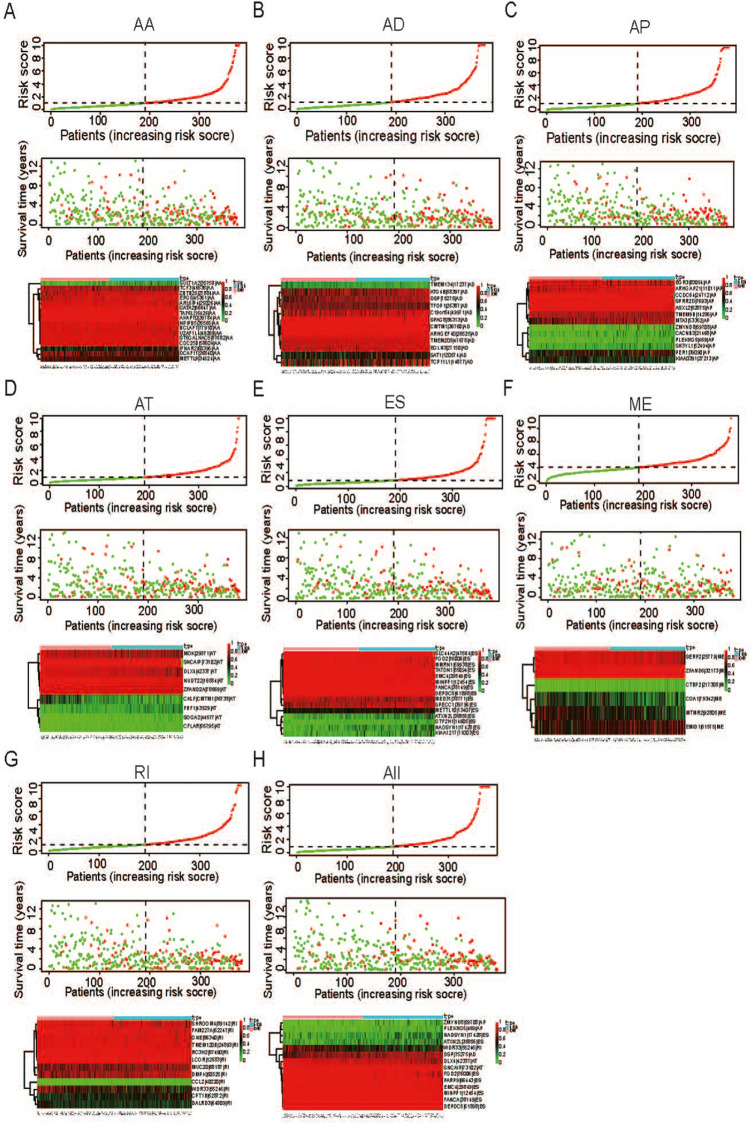
**a–h** Construction and analysis of risk scores of seven types of AS events and combined prognostic model. The top panels indicate the risk scores of the patients. The middle panels depict the survival status and survival time of patients distributed by risk score. The bottom panels display the heatmap of the PSI values for predictive factors distributed by risk score

The important prognostic-related AS events in all AS events in univariate Cox analysis were selected as candidates to select the most significant AS events by LASSO Cox regression model analysis (Fig. 3). Further, several prediction signatures based on these prognostic-associated AS events were constructed by multivariate Cox analyses. Eventually, a combined prognostic model was built integrated from different types of AS events (Table S2). As shown in Fig. 4a–h, Kaplan–Meier curves showed that LUSC patients in high-risk group had appreciably shorter OS than patients in low-risk group, demonstrating that these AS signatures could be powerful biomarkers to distinguish patients’ prognosis. Obviously, the combined prognostic indicator showed better performance than single type of AS events (Fig. 4h). Then, ROC curve was performed to appraise the prognostic efficiency of prognostic AS models. The results show that all signatures had a robust predictive property with AUC values from 0.837 to 0.978 (Fig. 4i). Conceivably, the combined model with all types of AS events had highest efficiency with 0.978 (AUC) than single prognostic models. Furthermore, we also explored the predictive effect of this combined prognostic model for RFS and found it had same powerful prognostic value and high accuracy (AUC = 0.793) in LUSC (Figure S1A-B). The distribution of patients’ risk score, survival status, and expression profiles of all AS models are shown in Fig. 5.

### The combined prognostic model exhibit better predictive ability than other model

We compared the predictive ability between our combined prognostic model and other model to verify the superiority of our model [26]. The Kaplan–Meier curve and ROC results showed that other mRNA had significantly predictive ability in LUSC, but our model had higher sensitivity and specificity than other mRNA model (Figure S2). Taken together, our combined prognostic model had superior prognostic value in LUSC patients.

### A network of prognosis-related AS genes and SFs

**Fig. 6:**
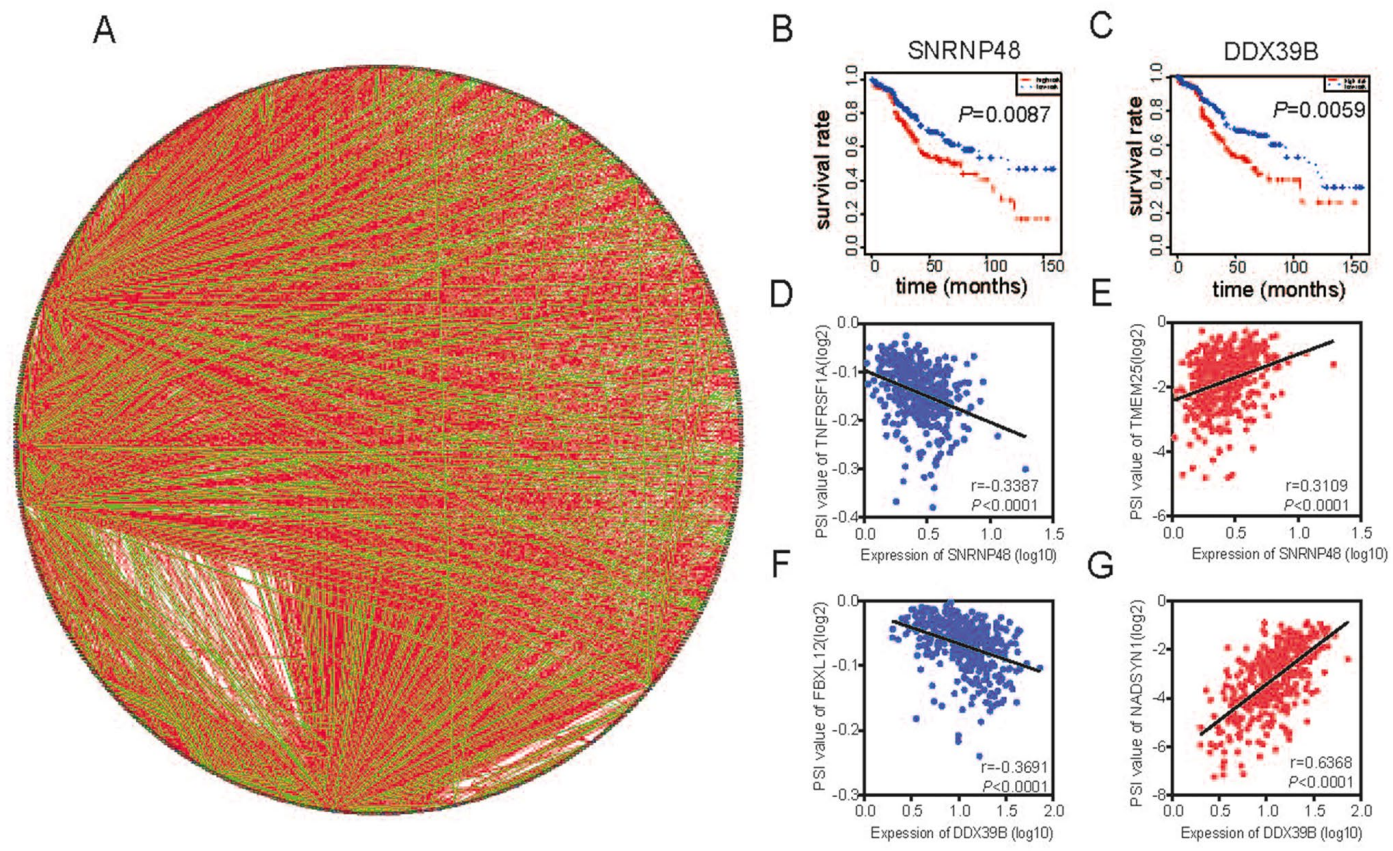
Splicing correlation network in LUSC. **a** Correlation network between expression of survival Splicing factors and PSI values of AS genes generated using Cytoscape. Purple dots were survival-associated splicing factors. Green/Red dots were favorable/adverse AS events. Red/Green lines represent positive/negative correlations between substances. **b–c** Kaplan–Meier curve of splicing factors SNRNP48 and DDX39B. **d–g** Representative dot plots of correlations between expression of splicing factors and PSI values of AS events

More importantly, extensive dysregulated AS events in many types of cancers are easily programmed by some specific SFs. Hence, an important issue is that whether several key SFs could potentially regulate these prognosis-associated AS events in LUSC. To determine those specific SFs which had close connection with the prognosis-associated AS events in LUSC, univariate Cox analysis of SFs were implemented according to gene expression level of LUSC patients. Consequently, there were 25 SFs obviously related to OS of LUSC patients shown in Table S3. Furthermore, correlations between SFs and prognostic AS events were tested in LUSC using Spearman’s test (Fig. 6a). In correlation networks, 22 SFs (purple dots) were obviously related to 546 prognosis-related AS events, including 202 favorable AS events (green dots) and 344 adverse AS events (red dots). Interestingly, there was a positive correlation (red lines) between majority of poor prognostic AS events (red dots) and SFs (purple dots), while there was a negative correlation (green lines) between majority of favorable prognostic AS events (green dots) and SFs. For example, splicing factors SNRNP48 and DDX39B had adverse survival for LUSC patients (Figs. 6b–c). ES of NADSYN1 and AP of TMEM25 were adverse factors, whereas AT of TNFRSF1A and AT of FBXL12 were related to favorable prognosis. Correlation between SNRNP48 and AT of TNFRSF1A or AP of TMEM25 were shown in dot plots, suggesting high expression of SNRNP48 had positive association with poor overall survival (Fig. 6d–e). Similarly, correlation between DDX39B and ES of NADSYN1 or AT of FBXL12 were shown in dot plots, implicating high expression of DDX39B had negative association with favorable prognosis (Fig. 6f–g).

## Discussion

Alternative splicing (AS) is an important process by which a single pre-mRNA precursor generates a large number of mature mRNAs and protein isoforms structurally and functionally [27, 28]. Accumulated evidence revealed that the plasticity of AS participated in cell metastasis, apoptosis, invasion, proliferation, immune evasion, and drug reliance of tumors by promoting cancer cells to produce isoform conversion [29–31]. So far, majority of studies focused on the exploration of AS events as biomarker for cancers have demonstrated that several AS events and spliced variants could be indicators to diagnose and predict cancers. For example, Kelley et al. revealed that the aberrant splicing expression of GSN gene had an obviously higher expression in tumor tissues than in adjacent tissues and regulated the HNSCC’s cell proliferation process [32]. Recently, a report suggested that KRAS-4A and KRAS-4B (KRAS isoforms) had significantly related to poor survival of CRC patients, especially microsatellite stable primary CRC [33]. Many studies demonstrated that CD44 could present multiple isoforms through variable mRNA splicing and CD44 isoforms play critical roles in tumor initiation and be considered as potential treatment target in CRC compared with CD44 [34, 35].

Currently, a lot of studies have also reported abnormal AS events that exerted crucial functions in lung cancer by promoting the initiation and progression of lung cancer. For example, a microarray study found differential splicing of MACF1, VEGFA, NUMB, and APP between NSCLC and control tissues in 29 patients [8]. In addition, AS variants were also related to drug sensitivity of lung cancer. It is previously reported that patients with lung adenocarcinoma with carcinogenic mutations at MET exon 14 RNA splice acceptor and donor sites could benefit from treatment with MET inhibitors crizotinib and cabozantinib, identifying a novel therapeutic target for lung adenocarcinoma [9]. Although some researchers have identified several prognostic alternative splicing events in LUAD and LUSC [7], with the development of high-throughput sequencing technique, novel prognosis-related AS events and potentially therapeutic targets needed to be explored further.

Here, we identified AS signatures and established regulatory network between AS events and SFs in LUSC through the analysis of TCGA program to gain systematic and comprehensive perception into RNA splicing patterns. In this study, a total of 1996 AS events were obviously related to LUSC patients' survival. Among the seven types of AS models, AD events showed the highest predictive power in survival prediction of LUSC patients than other six types of AS models. Moreover, we constructed a combined prognostic model composed of different splicing patterns of 14 genes including PLEKHG5, FANCA, ZMYND8 and so on. The combined prognostic model had higher predictive performance than any single type in seven AS models. In recent studies, some of these genes have been reported to exert carcinogenic or suppressive roles in cancers. For example, PLEKHG5 is a novel prognostic biomarker in glioma patients and could promote glioma migration and invasion [36]. FANCA was a prognostic factor in LUAD [37].

What's more, we identified some key SFs which might exert essential roles in the development and progression of cancers through modulating their corresponding AS events. With convincing results in this study that there was a positively correlation between most of poor prognosis-related AS events and SFs, while there was a negatively correlation between most of favorable prognosis-related AS events and SFs in LUSC, tt was well known that splicing factors could precisely regulate the splicing process by combining with specific genes’ the splice-regulatory sequence elements. This study provided an efficient approach to elucidate the underlying mechanism of AS events involved in survival of LUSC patients.

However, there are also some limitations in this study. Firstly, we could not verify the prognostic value of our combined prognostic model in other databases due to lack of available data. Secondly, the specific and valuable mechanisms of AS events and key SFs network need to be further explored. Finally, it is also need to further explore the interaction and regulation of AS events with other levels such as somatic mutation, copy number variation and DNA methylation.

In summary, we analyzed prognosis-related AS events and established AS signatures to predict survival of LUSC patients. Besides, an interesting splicing correlation network offered novel perceptions into how abnormal AS events were potentially modulated via pivotal SFs. These prognosis-related AS events and SFs provided us many valuable therapeutic targets for future validations and ultimately clarified the underlying mechanisms of AS in LUSC tumorigenesis.

## Supplementary Information

Below is the link to the electronic supplementary material.

Supplementary file1 (TIFF 109 kb)

Supplementary file2 (TIFF 140 kb)

Supplementary file3 (DOCX 13 kb)

Supplementary file4 (DOCX 17 kb)

Supplementary file5 (DOCX 18 kb)
