# Supplementary figures and images for: Development of alternative splicing signature in lung squamous cell carcinoma
Source: Med Oncol. 2021 Mar 27;38(5):49. doi: 10.1007/s12032-021-01490-1 (PMC8004499; doi:10.1007/s12032-021-01490-1)

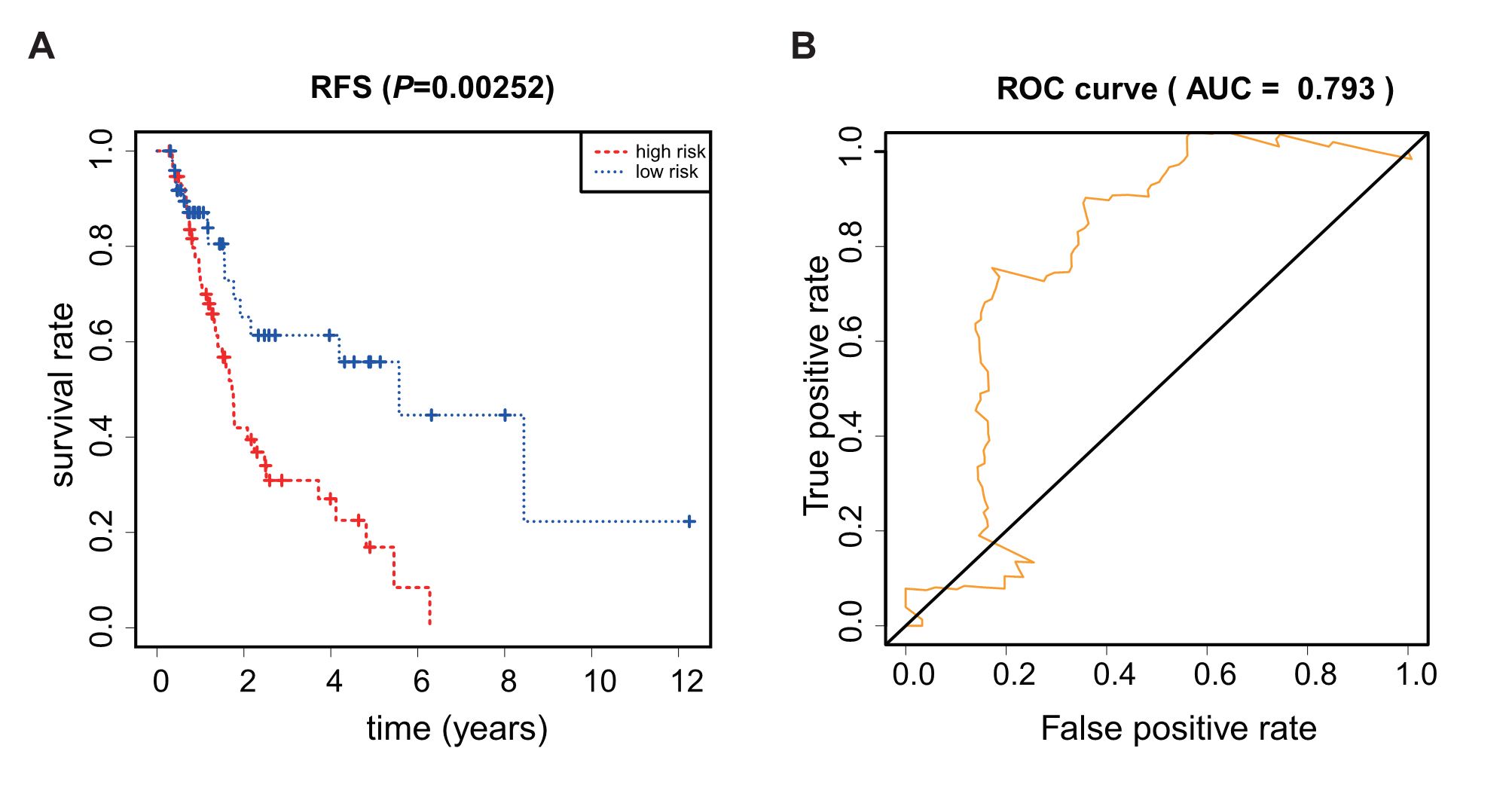

Supplement: Supplementary file 1 — Supplementary file1 (TIFF 109 kb) [file 12032_2021_1490_MOESM1_ESM.tiff]

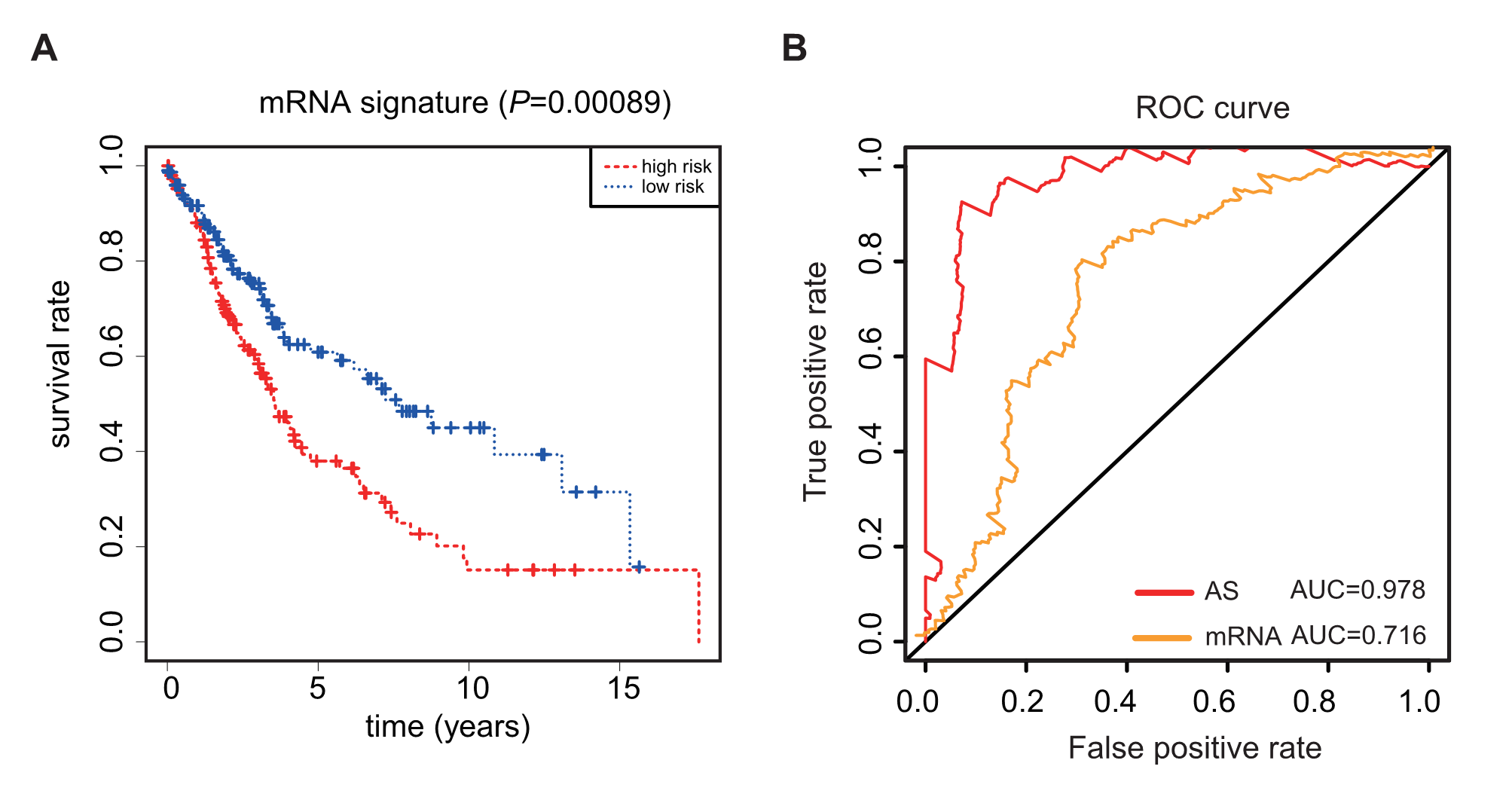

Supplement: Supplementary file 2 — Supplementary file2 (TIFF 140 kb) [file 12032_2021_1490_MOESM2_ESM.tiff]
