# Supplementary material for: Development of alternative splicing signature in lung squamous cell carcinoma
Source: Med Oncol. 2021 Mar 27;38(5):49. doi: 10.1007/s12032-021-01490-1 (PMC8004499; doi:10.1007/s12032-021-01490-1)
Supplement: Supplementary file 3 — Supplementary file3 (DOCX 13 kb) [file 12032_2021_1490_MOESM3_ESM.docx]

Table S1. Clinical characteristics of LUSC in TCGA data set

| characteristic | Entire series (%) |
| --- | --- |
| Gender |  |
| Male | 359/487(73.7) |
| Female | 128/487 (26.3) |
| Age(years) |  |
| >65 | 302/487(62.1) |
| ≤65 | 185/487(37.9) |
| Stage |  |
| I | 239/487 (49.2) |
| II | 159/487 (32.6) |
| III | 82/487(16.8) |
| IV | 7/487 (1.4) |
| Race |  |
| Non-white | 142/487(29.1) |
| White | 345/487(70.9) |
| T stage |  |
| T1 | 110/487 (22.6) |
| T2 | 286/487 (58.7) |
| T3 | 67/487 (13.8) |
| T4 | 24/487 (4.9) |
| N stage |  |
| N0 | 310/487 (63.7) |
| N1 | 128/487(26.3) |
| N2 | 38/487(7.8) |
| N3 | 11/487(2.2) |
| M stage |  |
| M0 | 402/487(82.5) |
| M1 | 85/487(17.5) |
