# Supplementary material for: Development of alternative splicing signature in lung squamous cell carcinoma
Source: Med Oncol. 2021 Mar 27;38(5):49. doi: 10.1007/s12032-021-01490-1 (PMC8004499; doi:10.1007/s12032-021-01490-1)
Supplement: Supplementary file 4 — Supplementary file4 (DOCX 17 kb) [file 12032_2021_1490_MOESM4_ESM.docx]

Table S2. The HR and *P* value of LUSC-all-multiCox

| Gene | Coefficient | HR | *P* value | Type |
| --- | --- | --- | --- | --- |
| DLX4 | -4.2089 | 0.0148(0.0010-0.2068) | 0.0017 | AT |
| DSP | 5.3175 | 23.8897(12.4115-33.4939) | 0.0002 | AD |
| FANCA | -15.0310 | 6.95e-66(1.43e-113-3.36e-18) | 0.0074 | ES |
| MINPP1 | -23.4904 | 6.28e-11(2.47e-16-1.60e-05) | 0.0002 | ES |
| WDR33 | -2.2665 | 0.1036(0.0308-0.3486) | 0.0003 | RI |
| ZMYND8 | 2.1700 | 8.7587(1.1146-68.8240) | 0.0391 | AP |
| NADSYN1 | 1.3435 | 3.8327(0.6667-22.0316) | 0.0132 | ES |
| ATXN2L | 2.1721 | 8.7773(1.7045-45.1985) | 0.0094 | ES |
| DEPDC5 | -16.5220 | 2.32e-64(1.10e-111-4.90e-17) | 0.0084 | ES |
| PARP9 | -7.5158 | 0.0005(4.68e-06-0.0633) | 0.0019 | ES |
| FGD2 | -6.8597 | 0.0010(2.63e-05-0.0418) | 0.0003 | ES |
| PLEKHG5 | 3.9325 | 51.0676(2.8083-927.5324) | 0.0079 | AP |
| SNCAIP | -3.0582 | 0.0469(0.0061-0.3560) | 0.0031 | AT |
| EMC4 | -10.6564 | 2.35e-05(1.08e-08-0.0513) | 0.0066 | ES |

HR, hazard ratio.
