# Supplementary material for: Development of alternative splicing signature in lung squamous cell carcinoma
Source: Med Oncol. 2021 Mar 27;38(5):49. doi: 10.1007/s12032-021-01490-1 (PMC8004499; doi:10.1007/s12032-021-01490-1)
Supplement: Supplementary file 5 — Supplementary file5 (DOCX 18 kb) [file 12032_2021_1490_MOESM5_ESM.docx]

Table S3. The HR and *P* value of LUSC-splicing factors

| Gene | HR(95%CI) | *P* value |
| --- | --- | --- |
| SNRNP48 | 4.2635(2.8860-6.2826) | 0.0008 |
| DDX39B | 2.5285(2.0357-3.1393) | 0.0009 |
| FUBP1 | 7.3517(4.7832- 11.2992) | 0.0104 |
| SNURF | 2.2989(1.2610- 4.1907) | 0.0111 |
| DHX15 | 7.8199(5.0279- 12.1621) | 0.0113 |
| DDX17 | 9.0146(6.1564- 13.1997) | 0.0134 |
| CCDC94 | 0.2275(0.1544- 0.3350) | 0.0141 |
| DHX38 | 4.3052(2.9206- 6.3461) | 0.0159 |
| ZC3H18 | 4.4908(2.8384-7.1050) | 0.0173 |
| INTS3 | 3.3445(2.3345-4.7913) | 0.0192 |
| AQR | 3.7458(2.3235-6.0385) | 0.0222 |
| C19orf43 | 0.1181(0.0805-0.1732) | 0.0229 |
| ZC3H13 | 3.6101(2.3561-5.5313) | 0.0248 |
| CDK11A | 1.8986(1.2894-2.7955) | 0.0288 |
| TTC14 | 1.9117(1.5136-2.4144) | 0.0297 |
| MBNL2 | 2.2723(1.7725-2.9129) | 0.0320 |
| CLK2 | 3.1146(2.2821-4.2507) | 0.0340 |
| U2SURP | 2.7915(2.1066-3.6989) | 0.0348 |
| BUD31 | 0.2293(0.1603-0.3278) | 0.0381 |
| RBM39 | 4.3864(3.0479-6.3126) | 0.0384 |
| RNPC3 | 2.1898(1.3731-3.4922) | 0.0389 |
| SRSF6 | 7.0384(4.5569-10.8710) | 0.0448 |
| CDK10 | 2.3355(1.6956-3.2168) | 0.0474 |
| DDX46 | 4.2749(2.5322-7.2169) | 0.0484 |
| TXNL4A | 0.3241(0.2209-0.4754) | 0.0494 |

HR, hazard ratio.
